# Deep Brain Stimulation Effects on Gait Pattern in Advanced Parkinson’s Disease Patients

**DOI:** 10.3389/fnins.2020.00814

**Published:** 2020-08-14

**Authors:** Daniela Navratilova, Alois Krobot, Pavel Otruba, Martin Nevrly, David Krahulik, Petr Kolar, Barbora Kolarova, Michaela Kaiserova, Katerina Mensikova, Miroslav Vastik, Sandra Kurcova, Petr Kanovsky

**Affiliations:** ^1^Department of Neurology, University Hospital and Faculty of Medicine and Dentistry, Palacký University Olomouc, Olomouc, Czechia; ^2^Department of Rehabilitation, University Hospital and Faculty of Medicine and Dentistry, Palacký University Olomouc, Olomouc, Czechia; ^3^Department of Neurosurgery, University Hospital and Faculty of Medicine and Dentistry, Palacký University Olomouc, Olomouc, Czechia

**Keywords:** deep brain stimulation, subthalamic nucleus, gait, pressure-sensitive treadmill, biomechanical parameters of gait

## Abstract

**Background:**

Gait disturbance accompanies many neurodegenerative diseases; it is characteristic for Parkinson’s disease (PD). Treatment of advanced PD often includes deep brain stimulation (DBS) of the subthalamic nucleus. Regarding gait, previous studies have reported non-significant or conflicting results, possibly related to methodological limitations.

**Objective:**

The objective of this prospective study was to assess the effects of DBS on biomechanical parameters of gait in patients with PD.

**Methods:**

Twenty-one patients with advanced PD participated in this prospective study. Gait was examined in all patients using the Zebris FDM-T pressure-sensitive treadmill (Isny, Germany) before DBS implantation and after surgery immediately, further immediately after the start of neurostimulation, and 3 months after neurostimulator activation. We assessed spontaneous gait on a moving treadmill at different speeds. Step length, stance phase of both lower limbs, double-stance phase, and cadence were evaluated.

**Results:**

In this study, step length increased, allowing the cadence to decrease. Double-stance phase duration, that is, the most sensitive parameter of gait quality and unsteadiness, was reduced, in gait at a speed of 4.5 km/h and in the narrow-based gaits at 1 km/h (tandem gait), which demonstrates improvement.

**Conclusion:**

This study suggests positive effects of DBS treatment on gait in PD patients. Improvement was observed in several biomechanical parameters of gait.

## Introduction

Gait is a unique, extraordinarily complex motor behavior that is controlled by multiple areas of the central nervous system at the corticosubcortical and brainstem levels. Physiological gait can be achieved through the flawless coordination of three primary components: (1) locomotion, (2) balance, and (3) ability to adapt to the environment (Snijders et al., 2007). Gait is also influenced by cognitive and psychological states. Any dysfunction in the musculoskeletal or nervous system can lead to gait alteration.

The feared consequences of gait disturbance are usually falls and subsequent injuries. Disease progression, injuries, and the fear of falling lead to declines in walking ability and increased patient dependency, that is, reliance on the help of others. Impaired mobility or immobility results in insufficient exposure to stimuli from the external environment and often leads to loss of patient interest in the environment and social isolation (Snijders et al., 2007).

The prevalence of gait disturbance increases with age. It is common in the elderly. Fifteen percent of people aged 60–84 years’ experience gait disturbance; it affects 82% of individuals 85 years or older (Snijders et al., 2007). Gait disturbance accompanies many neurodegenerative diseases; it is characteristic for Parkinson’s disease (PD), especially at the stage of late motor complications – short steps, hesitations, and sometimes freezing. Gait is further influenced by hypokinesia, bradykinesia, postural instability, and non-motor symptoms such as cognitive deficit, psychological state, camptocormia. Because of these motor and non-motor symptoms, falls with the above stated consequences may happen in patients with PD.

Treatment of advanced PD often includes deep brain stimulation (DBS) of the subthalamic nucleus, which improves the motor deficit, especially bradykinesia and rigidity (Lilleeng et al., 2015). Regarding gait, previous studies have reported non-significant or conflicting results (Pötter-Neger and Volkmann, 2013; St George et al., 2014; Collomb-Clerc and Welter, 2015), possibly related to methodological limitations of gait evaluation using questionnaire methods, the Timed Up and Go Test or employing sensors attached to patients’ lower limbs and trunk, where varying speed in subsequent gait tests distorts the results and comparisons.

The objective of this prospective study was to elucidate the biomechanical parameters (step length, stance phase, double-stance phase, cadence) of gait in advanced PD patients and to quantify their possible changes after implantation of the subthalamic nucleus (STN) with DBS. In contrast to previous works on this topic (Pötter-Neger and Volkmann, 2013; St George et al., 2014; Collomb-Clerc and Welter, 2015), in this study, a new measurement method was applied using the Zebris FDM-T pressure-sensitive treadmill (Isny, Germany) that made it possible to analyze pressure distribution under the feet while standing and walking (N/cm^2^). Pressure (contact) each foot applied on treadmill belt is observed in space and in time and immediately recorded. Thanks to this rigorous registration of limb pressure inception and duration, the system is able to calculate step length (distance between onset of pressure exerted by one foot to first registration of pressure by the other foot), stance phase (time of duration pressure of one foot on belt), double-stance (time of duration of pressure both feet at one moment), and cadence (number of pressure events per minute).

Moreover, the correlation between the biomechanical gait parameters and patient age and disease duration was investigated in this prospective study. These correlations have not yet been satisfactorily examined in movement disorder studies.

## Materials and Methods

Twenty-one patients were enrolled prospectively in this study between March 2015 and December 2018. One male patient was excluded because of severe postural instability and inability to finish the last measurement. In total, statistical analysis was carried out on 20 patients (16 males, 4 females, aged 48–71 years, median 62 years, SD 5.84 years) with advanced PD at the stage of late motor complications (on–off fluctuation, wearing off, peak of dose dyskinesia, off dyskinesia of the character of dystonia) who met the criteria for surgery and underwent bilateral STN-DBS implantation. The duration of the disease has been on average 8.11 years (median 8.0 years, SD 3.19 years); for complete information (see Table 1). Unified Parkinson’s Disease Rating Scale (UPDRS) III preoperatively in the ON medication state (baseline) was 17.1 on average, SD 5.66; and UPDRS IV 4.0 on average, SD 1.34. For complete information (see Table 2). The patients did not suffer from dyskinesias preventing or significantly limiting walking during the gait testing with the Zebris FDM-T (Isny). All the patients had levodopa-responsive gait difficulties before surgery. The PD motor symptoms were present bilaterally in the patients. Only one patient walked with the support of two French sticks due to back pain. No one else had another disease affecting walking. Genetic testing was performed on one patient younger than 50 years because of the presence of PD in the family, but the result is not available. In the Czech Republic, genetic testing is not routinely performed.

**TABLE 1:** Demographic data and medication.

Patient	Sex	Age/years	Duration of disease/years	Daily dose of levodopa/Dopamine agonists preoperatively and immediately after surgery in mg	Daily dose of levodopa/Dopamine agonists 1 and 3 months postoperatively in mg	Side prevalence of the disease
1	M	60	10	1250/0	600 mg/0	left
2	M	71	13	1200/24	750/0	right
3	F	67	3	300/16	200/8	left
4	F	48	5	143.5/2.8	550/2.1	right
5	M	57	10	1450	750	right
6	M	62	5	1250/8	500/8	right
7	M	58	3	200/8	0/0	right
8	M	65	8	750/2.1	625/0	left
9	M	57	8	650/24	400/8	right
10	F	63	9	500/24	400/8	left
11	M	69	5	750/12	400/8	left
12	M	65	10	600/12	600/8	right
13	M	62	5	600/24	400/8	left
14	M	49	6	700/20	0/12	left
15	M	66	9	750/2.1	400/2.1	left
16	F	56	8	400/20	0/12	right
17	M	59	13	1000/16	625/8	right
18	M	62	14	800/16	400/8	right
19	M	61	10	400/8	0/12	left
20	M	59	9	1125/0	500/0	left

**TABLE 2:** UPDRS scale.

Patient	UPDRS III preoperatively/ON medication state	UPDRS III 3 months after DBS activation/ON medication state	UPDRS IV preoperatively/ON medication state	UPDRS IV 3 months after DBS activation/ON medication state
1	25	18	4	2
2	20	4	4	1
3	10	6	5	3
4	12	7	3	2
5	25	20	4	3
6	11	8	4	1
7	18	7	5	0
8	13	14	4	3
9	12	5	4	1
10	15	8	5	1
11	21	13	2	0
12	15	11	8	1
13	20	18	3	4
14	14	8	4	2
15	10	7	3	3
16	15	6	2	0
17	33	20	3	2
18	18	16	3	0
19	18	11	6	3
20	17	12	4	2
Average	17.1	10.95	4	1.7
DS	5.66	5.04	1.34	1.18

The examination of biomechanical parameters of gait was carried out after obtaining written informed consent from all patients. The measurement was conducted using the Zebris FDM-T System (Isny) that analyzes pressure distribution under the feet while standing, walking, or running on a treadmill (N/cm^2^). Examinations were scheduled (1) before DBS implantation, (2) after electrode implantation (i.e., 3–5 days after surgery), (3) after neurostimulator activation (i.e., 1 month after surgery), and (4) at 3 months after DBS activation.

We assessed spontaneous gait, that is, without “coaching” by a therapist, on a moving treadmill at speeds of 1.5, 3, and 4.5 km/h and subsequently the narrow-based gait at speeds of 1 and 2.5 km/h. Gait parameters are directly dependent on gait speed. Thus, the speed was exactly defined with regard to reproducibility. The speed was chosen on the basis of empirical experience. The speed PD patients find comfortable is approximately 3 km/h. The slow speed of 1 km/h was chosen because it causes great problems to PD patients. They have to concentrate more on walking and maintaining stability. Contrasting with the optimal speed (i.e., 3 km/h), the fast gait speed of 4.5 km/h is chosen in order to accentuate deviations in gait parameters. The narrow-based gait, or tandem gait, is generally more demanding with respect to coordination, stability. Thus, two speeds were chosen in order to find out whether gait parameters are changed under these demanding conditions. The patients were walking 1 min at each speed in the training mode, and the measurement of parameters was carried out continuously in the second minute. There was a 2 min break between the changes to rest.

The patients were examined in the ON medication state every time, after application of their standard medication dose. Supradose was not administered. The third and the fourth measurements were performed with the DBS system involved, parameters set on 60 μs pulse duration, and 130 Hz pulse frequency, voltage was individual in each case according to clinical effect usually about 3.0 V. Medication was adjusted together with the DBS. Deep brain stimulation was optimally set at 3 months after activation.

In each examination, the following biomechanical parameters of gait were evaluated:

• Step length of the right lower limb, step length of the left lower limb (i.e., the distance between the heel in contact with the treadmill belt on one side and the heel in contact with the belt on the contralateral side, measured in cm);
• Stance phase of the right lower limb and of the left lower limb (i.e., duration of the gait cycle phase in which the contralateral lower limb is not in contact with the treadmill belt, expressed in percent);
• Double-stance phase (i.e., duration of the gait cycle phase in which both lower limbs are in contact with the treadmill belt, expressed in percent); and
• Cadence (i.e., number of steps per minute – calculated from the stride time, which is the duration of a gait cycle between the contact of one heel and the consecutive contact of the same heel with the treadmill belt expressed in seconds).

In statistical analysis, we tested whether there is a correlation between measured biomechanical gait parameters and proband’s age and duration of disease. Patients were divided into two groups of 48–60 years (i.e., 9 subjects, mean age 55.89 years, SD 4.12 years) and 61–71 years (i.e., 11 probands, mean age 64.82 years, SD 3.07 years) and according to the duration of the disease into two groups: (i) up to 9 years of disease duration (average length 6.17 years, SD 2.07 years) and (ii) from 10 years upward (average length 11.25 years, SD 1.64 years). The patient sample was divided into groups on the basis of its composition so that a similar number of patients was in each comparison group.

In the statistical data analysis, normality was first verified using the Shapiro–Wilk test. Next, the results were evaluated using repeated-measures analysis of variance with age and sex as covariates. Then the Tukey honestly significant difference test was applied as a *post hoc* test. Dependence of gait parameters and the Parkinsonian features, evaluated by means of UPDRS III, was tested with Spearman correlation coefficient.

Data were analyzed using SW STATISTICA, version 12 (StatSoft, Tulsa, OK, United States).

With the exception of step length of the left lower limb in gait at the speed of 4.5 km/h, step length of the right lower limb at the speed of 3 km/h, at the speed 4.5 km/h, and at the speed of 2.5 km/h (narrow-based gait), cadence at the speed of 1.5 km/h, the results were not significant.

The significance level was set to 0.05.

## Results

The analysis of the step length yielded the following results: in summary, step length of the left lower limb increased following the DBS treatment 3 months after activation (at a gait speed of 1.5 km/h by 7.99 cm, *p* = 0.24; at a gait speed of 3 km/h by 2.53 cm, *p* = 0.2; at a gait speed of 4.5 km/h by 2.23 cm, *p* = 0.004; in the narrow-based gaits at a speed of 1 km/h by 5.61 cm, *p* = 0.42; and in the narrow-based gaits at a speed of 2.5 km/h by 0.52 cm, *p* = 0.07). Likewise, step length of the right lower limb increased following the DBS treatment 3 months after activation (at a gait speed of 1.5 km/h by 0.61 cm, *p* = 0.44; at a gait speed of 3 km/h by 4.07 cm, *p* = 0.04; at a gait speed of 4.5 km/h by 1.94 cm, *p* = 0.04; in the narrow-based gaits at a speed of 1 km/h by 4.81 cm, *p* = 0.45; and in the narrow-based gaits at speed 2.5 km/h by 5.36 cm, *p* = 0.04). For more details (see Table 3).

**TABLE 3:** Biomechanical parameters of gait.

Type of gait	1.5 km/h					
	
	Before DBS	After electrode implantation	After neurostimulator activation	3 months after activation	*p*	Partial η^2^
Step length left	27.28 (10.88)	36.28 (17.52)	33.40 (12.05)	35.27 (8.43)	0.24	0.09
Step length right	34.40 (18.28)	36.10 (21.52)	36.84 (11.46)	35.01 (8.49)	0.44	0.07
Stance phase duration left	68.95 (3.41)	68.46 (4.44)	69.66 (3.69)	69.75 (3.08)	0.32	0.07
Stance phase duration right	68.97 (3.72)	68.59 (3.16)	69.08 (4.81)	69.34 (3.81)	0.84	0.02
Double-stance phase	38.25 (4.71)	37.70 (6.76)	39.46 (7.59)	39.11 (6.08)	0.59	0.05
Cadence	90.25 (20.59)	75.90 (18.32)	72.85 (15.96)	73.26 (16.94)	**0.002**	0.27

**Type of gait**	**3 km/h**					
	
	**Before DBS**	**After electrode implantation**	**After neurostimulator activation**	**3 months after activation**	***p***	**Partial η^2^**

Step length left	46.15 (5.96)	47.7 (6.73)	49.45 (7.21)	48.68 (7.42)	0.20	0.01
Step length right	44.35 (6.07)	45.75 (8.33)	48.35 (7.73)	48.42 (6.63)	**0.04**	0.16
Stance phase duration left	62.94 (4.95)	64.20 (2.19)	64.44 (2.97)	64.29 (2.66)	0.66	0.03
Stance phase duration right	63.96 (5.94)	64.97 (3.21)	63.89 (3.56)	65.02 (1.96)	0.56	0.04
Double-stance phase	29.26 (4.08)	29.14 (4.50)	28.08 (5.67)	29.24 (3.93)	0.15	0.11
Cadence	109.40 (14.07)	104.40 (18.64)	104.80 (16.47)	105.32 (15.91)	0.47	0.05

**Type of gait**	**4.5 km/h**					
	
	**Before DBS**	**After electrode implantation**	**After neurostimulator activation**	**3 months after activation**	***p***	**Partial η^2^**

Step length	59.53 (6.03)	58.68 (7.26)	61.2 (7.49)	61.76 (7.79)	**0.00**	0.31
Step length right	59.00 (6.25)	58.47 (8.17)	60.90 (8.01)	60.94 (7.49)	**0.04**	0.20
Stance phase duration left	61.71 (2.21)	61.83 (2.45)	62.06 (2.34)	61.83 (2.67)	0.80	0.03
Stance phase duration right	62.38 (2.20)	62.34 (2.65)	61.77 (2.53)	61.83 (2.68)	**0.04**	0.21
Double-stance phase	24.06 (3.58)	24.37 (4.20)	23.80 (4.52)	23.66 (5.03)	0.12	0.15
Cadence	124.47 (13.12)	124.00 (13.26)	123.15 (15.5)	123.29 (17.25)	0.58	0.05

**Type of gait**	**Narrow-based gait 1 km/h**					
	
	**Before DBS**	**After electrode implantation**	**After neurostimulator activation**	**3 months after activation**	***p***	**Partial η^2^**

Step length left	38.33 (8.21)	41.4 (14.42)	40.25 (5.92)	43.94 (21.62)	0.42	0.10
Step length right	36.66 (12.69)	33.87 (15.58)	41.65 (6.98)	41.47 (21.2)	0.45	0.08
Stance phase duration left	72.33 (7.22)	72.82 (8.12)	74.56 (5.93)	72.53 (6.15)	0.55	0.07
Stance phase duration right	72.44 (6.91)	71.69 (8.01)	74.25 (7.07)	72.52 (4.70)	0.56	0.07
Double-stance phase	65.24 (64.77)	78.86 (99.63)	47.76 (13.10)	51.73 (15.24)	0.52	0.08
Cadence	43.11 (13.58)	43.53 (12.36)	43.45 (6.78)	40.71 (4.58)	0.10	0.17

**Type of gait**	**Narrow-based gait 2.5 km/h**					
	
	**Before DBS**	**After electrode implantation**	**After neurostimulator activation**	**3 months after activation**	***p***	**Partial η^2^**

Step length left	49.11 (8.25)	47.71 (8.58)	48.42 (7.58)	49.63 (7.86)	0.07	0.17
Step length right	49.05 (8.09)	44.84 (13.70)	48.25 (6.65)	49.84 (8.66)	**0.04**	0.17
Stance phase duration left	64.81 (2.91)	65.15 (2.75)	65.41 (2.62)	64.85 (2.38)	0.42	0.64
Stance phase duration right	65.30 (3.20)	64.99 (2.17)	65.57 (3.14)	65.42 (3.67)	0.71	0.32
Double-stance phase	29.61 (4.97)	40.52 (39.94)	31.47 (4.89)	32.38 (10.99)	0.51	0.06
Cadence	85.26 (14.04)	88.89 (13.41)	86.90 (13.59)	86.00 (13.68)	0.28	0.00

*The table shows average values and corresponding standard deviations in brackets. Significant differences are marked in bold font. Step length is given in centimeters, stance phase duration and double-stance phase in % of the total duration of the gait cycle, and cadence in steps per minute.*

The stance phase duration of the left lower limb: the stance phase duration did not change in the left lower limb as expressed in percent of the gait cycle 3 months after activation. The analysis of stance phase duration of the right lower limb: in summary, the stance phase duration did not change in the right lower limb as expressed as a percent of the gait cycle 3 months after activation, except of the gait at a speed of 4.5 km/h, when stance phase duration significantly decreased from 62.38 to 61.83%, *p* = 0.04.

In the analysis of double-stance phase, expressed as a percent, the following results were obtained: overall, double-stance phase duration decreased in gait at a speed of 4.5 km/h (from 24.06 to 23.66%, *p* = 0.12) and in the narrow-based gaits at 1 km/h (from 65.24 to 51.73%, *p* = 0.52) 3 months after activation.

The cadence analysis yielded the following results: in general, cadence decreased (at a gait speed of 1.5 km/h by 16.9 steps/min, *p* = 0.002; at a gait speed of 3 km/h by 4.08 steps/min, *p* = 0.47; at a gait speed of 4.5 km/h by 1.18 steps/min, *p* = 0.58; and in the narrow-based gaits at a speed of 1 km/h by 2.4 steps/min, *p* = 0.1) with the exception of the narrow-based gait at a speed of 2.5 km/h (when cadence increased by 0.74 steps/min, *p* = 0.28) 3 months after activation.

The analysis of measurements immediately after electrode implantation revealed the following: the step length in both the right and left lower limbs increased at the gait speed of 3 km/h (by 1.55 cm in the left lower limb and 1.4 cm in the right one) and 1.5 km/h (by 9 cm in the left one and 1.7 cm in the right one); the step length was shortened in both the lower limbs at the gait speed of 4.5 km/h (by 0.85 cm in the left one and 0.53 cm in the right one) and the narrow-based gait (at a speed of 1 km/h by 2.79 cm in the right one, at a speed of 0.5 km/h by 1.4 cm in the left one and by 4.21 cm in the right one). The stance phase remained unchanged bilaterally. The double-stance phase shortened (at a gait speed of 1.5 km/h by 0.55%, at a gait speed of 3 km/h by 0.12%) with the exception of gait at 4.5 km/h (it lengthened by 0.31%) and the narrow-based gait at 1 km/h (it lengthened by 13.62%) and 2.5 km/h (it lengthened by 0.91%). The overall cadence decreased too (at a gait speed of 1.5 km/h by 14.35 steps/min, at a gait speed of 3 km/h by 5 steps/min, at a gait speed by 0.47 steps/min), with the exception of the narrow-based gait (it increased by 0.34 steps/min at a speed of 1 km/h and by 3.63 steps/min at a gait speed of 2.5 km/h).

The analysis of measurements immediately after neurostimulator activation yielded the following results: the step length increased in both the right and left lower limbs (right – at a gait speed of 1.5 km/h by 2.42 cm, at a gait speed of 3 km/h by 4.0 cm, at a gait speed of 4.5 km/h by 1.9 cm, in the narrow-based gaits at a speed of 1 km/h by 4.99 cm, left – at a gait speed of 1.5 km/h by 6.12 cm, at a gait speed of 3 km/h by 3.30 cm, at a gait speed of 4.5 km/h by 1.67 cm, and in the narrow-based gaits at a speed of 1 km/h by 1.92 cm) with the exception of the narrow-based gait at the speed of 2.5 km/h (shortening by 0.8 cm in the right one and by 0.69 cm in the left one). The stance phase increased in both the lower limbs (by 0.95% on average, DS 0.74). The double-stance phase decreased (at a gait speed of 3 km/h by 1.18%, at a gait speed of 4.5 km/h by 0.26%, in the narrow-based gait at a speed of 1 km/h by 17.48%) with the exception of gait speed of 1.5 km/h (lengthening by 1.21%) and the narrow-based gait at the speed of 2.5 km/h (by 1.86%). The cadence decreased (at a gait speed of 1.5 km/h by 17.4 steps/min, at a gait speed of 3 km/h by 4.6 steps/min, at a gait speed of 4.5 km/h by 1.32 steps/min) with the exception of the narrow-based gait (at a gait speed of 1 km/h, an increase by 0.34 steps/min, and at a gait speed of 2.5 km/h by 1.64 steps/min).

For complete results (see Table 3).

**FIGURE 1:**
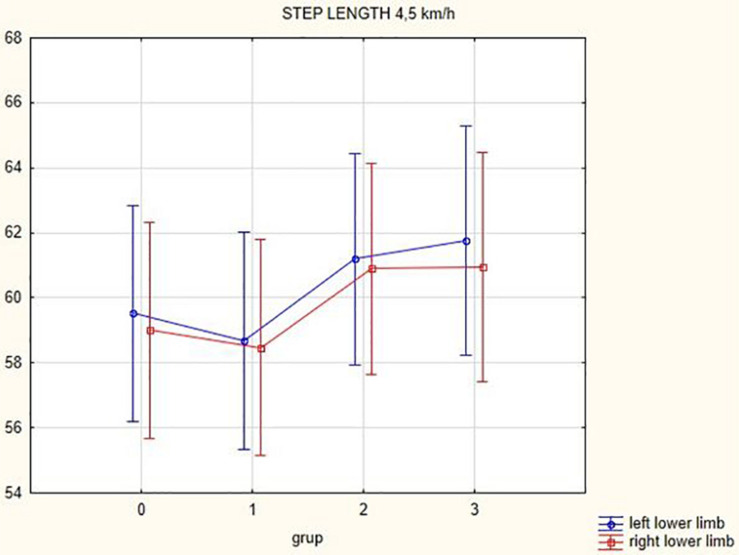
Step length of the left and the right lower limb in the gait at a speed of 4.5 km/h.

**FIGURE 2:**
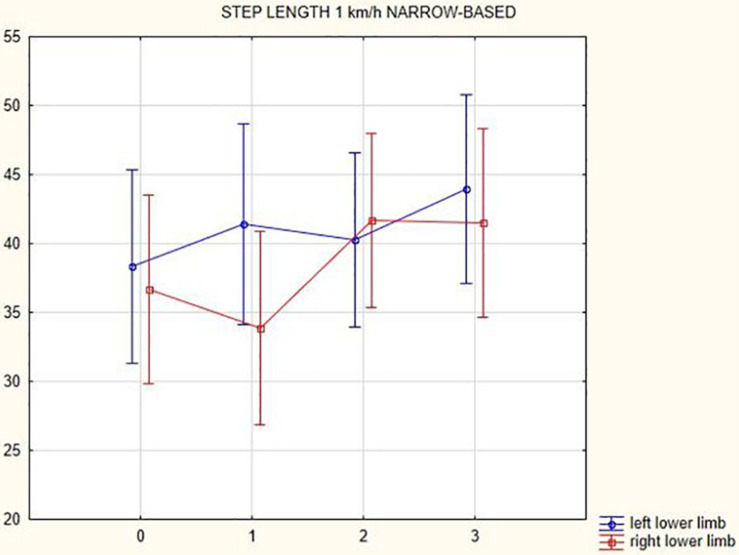
Step length of the left and the right lower limb in the narrow-based gait at a speed of 1 km/h.

**FIGURE 3:**
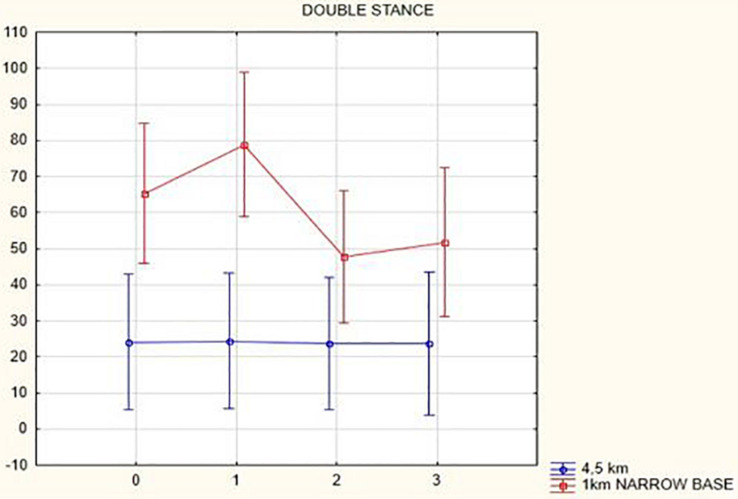
Double stance phase duration in the gait at a speed 4.5 km/h and in the narrow-based gait at a speed of 1 km/h.

**FIGURE 4:**
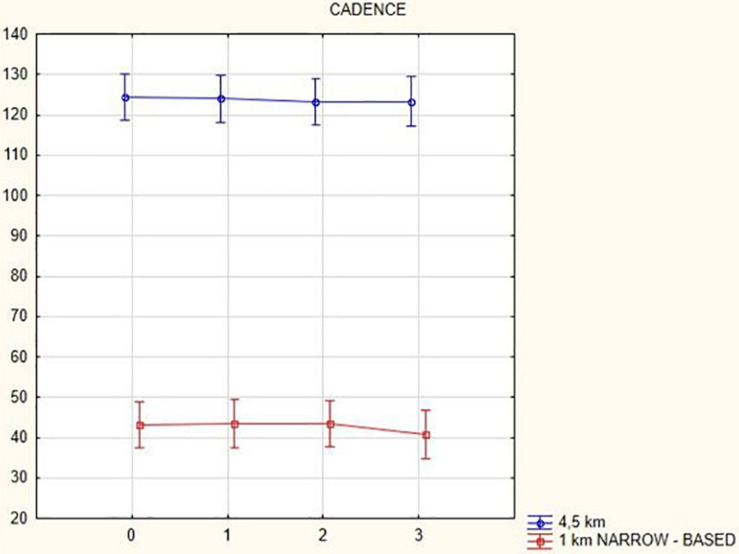
Cadence in the gait at a speed 4.5 km/h and in the narrow-based gait at a speed of 1 km/h.

Figures 1, 2 illustrate biomechanical parameters during gait at speed of 4.5 km/h and in the narrow-based gait at a speed of 1 km/h, which are the most demanding for PD patients. Figure 3 shows double-stance phase duration, the most sensitive parameter of gait quality and unsteadiness. Figure 4 shows cadence over time, which changes inversely to step length.

We did not find a statistically significant correlation between the measured biomechanical gait parameters and patient age and disease duration.

When comparing the average values of the UPDRS III scale preoperatively in the ON medication state and 3 months after the start of stimulation with an activated stimulator and in the ON medication state simultaneously, there was a decrease in the score from the average of 17.1 (SD 5.66) to 10.97 (SD 5.04). When comparing UPDRS IV, a decrease from the average of 4 (SD 1.34) to 1.7 (SD 1.18) occurred. For complete results (see Table 2).

No significant correlation between UPDRS III and gait parameters before DBS implantation was found, not even when correlating values from examination 3 months after DBS activation, with the exception of stance phase.

There is a correlation between the UPDRS III scale and stance phase duration of the right lower limb of gait at speed 3 km/h before DBS implantation (the significant correlation coefficient is 0.54, *p* = 0.01) and between UPDRS III and stance phase duration of the left lower limb for narrow-based gait at speed 1 km/h when examined 3 months after DBS activation (the significant correlation coefficient is −0.54, *p* = 0.03). There is no correlation between UPDRS III and stance phases at other speeds or in other gait parameters.

## Discussion

In this prospective study, we observed improved biomechanical parameters of gait associated with DBS treatment. During gait at speed of 4.5 km/h and during narrow-based gait at speed of 1 km/h, which are the most demanding for PD patients, we observed that step length of both lower limbs increased at 3 months after DBS activation, when assessed using a Zebris treadmill (Figures 1, 2). Most importantly, double-stance phase duration (Figure 3), i.e., the most sensitive parameter of gait quality and unsteadiness, was reduced, in both cases. Because of the increased step length, cadence decreased (Figure 4). With the exception of step length of the left lower limb in gait at speed 4.5 km/h, step length of the right lower limb at the speed of 3 km/h, at the speed 4.5 km/h, and at the speed of 2.5 km/h (narrow-based gait) and cadence at the speed of 1.5 km/h, the results were not significant. However, the clinical significance, as can be seen in the figures, suggests that DBS treatment has a more positive impact on gait than the medication alone.

Overall improvement in gait is found during gait examination immediately after DBS activation. The greatest problems were caused by the narrow-based gait. We identify a limitation of this examination in a short time after DBS activation when the patients have not completely adapted, and further fine-tuning of the stimulation parameters was done during outpatient checkups. This was why gait was also examined 3 months after DBS activation.

The gait examination immediately after electrode implantation in the ON medication state (when the total daily levodopa dose remains the same as it was before surgery) without activated DBS shows deterioration of gait under demanding circumstances, for example, fast gait or narrow-based gait. We ascribe this deterioration to postoperative fatigue.

The only isolated correlation emerged between UPDRS III and the stance phase of the right lower limb at the speed of 3 km/h before surgery when this parameter grew with the increasing UPDRS III; this shows that the patient loaded the right lower limb for a longer time when walking. Surprisingly, the stance phase of the left lower limb got shorter during narrow-based gait at the speed of 1 km/h 3 months after surgery. No correlation was found between other gait parameters and UPDRS III, probably due to the fact that the UPDRS III questionnaire is very complex and includes many Parkinsonian symptoms. It can only be stated that there is an indication of shortening step length in the left and right lower limbs when the UPDRS III value grows and cadence increases.

The relevance of our findings is supported by the methodological approach used. In the present study, we did not rely on less valid questionnaire scales; we acquired prospective data employing a Zebris system consisting of a dynamic belt with integrated pressure sensors and a computer measurement system connected to the treadmill belt. Measurement can be monitored in real time. The computer system subsequently creates measurement reports for specific biomechanical parameters of gait in the form of figures and tables. Whereas biomechanical parameters are changed according to speed of gait, investigation at exactly defined speed of gait makes it possible to precisely compare biomechanical parameters with the follow-up examinations (for example, after DBS implantation). In contrast, studies comparing gait without predefined speed during walking on the floor (with sensors on limbs) yield biomechanical parameters that are not so well comparable.

We included step length in the analysis of biomechanical parameters because the step length is shortened in PD and because it is the basic gait parameter. Double-stance phase and stance phase were evaluated because they indicate stability and confidence. Cadence indicates how many steps the patients make per minute. Generally, cadence increases as the step length decreases.

The effect of DBS treatment has been evaluated in other available published works using the questionnaire-based motor scale UPDRS III, the Timed Up and Go Test, and the dual-task ability test (Deuschl et al., 2013; Mera et al., 2013; St George et al., 2014; Vercruysse et al., 2014; Lilleeng et al., 2015; Collomb-Clerc and Welter, 2015; Vallabhajosula et al., 2015; Lizarraga et al., 2016; Brandmeir et al., 2016; Lei et al., 2016). There are also meta-analyses and studies reporting some biomechanical parameters of gait (step length and cadence), although only marginally and without any description of the measurement methods used (Pötter-Neger and Volkmann, 2013). Similar to Pötter-Neger and Volkmann (2013), we detected an increase in the stride length during the DBS treatment, however, contrary to their findings, we observed a decrease in cadence in our study. A study of Lizarraga et al. (2016) evaluated gait kinematics, step length, and gait speed using sensors placed on ankles during a “stand-walk-and-sit” test. In line with our findings, step length was seen to increase. Speed has also been reported to improve, which was not evaluated in this study because the velocity was precisely defined by the moving treadmill. Gait parameters measured using a Zebris treadmill are more accurate because step length depends on walking speed. The study of Lizarraga et al. (2016) reported no significant differences in kinematic parameters during unilateral and bilateral STN stimulation.

In one study of Mera et al. (2013), the tasks included in UPDRS III were kinematically evaluated using five sensors (three sensors to measure linear acceleration and two for angular velocity) along with the UPDRS III score. The sensor evaluation was seen as more accurate than the scale. What we see as a limitation of this study when gait was evaluated technically by means of sensors in a more precise way (twice in patients with DBS) is the inability to repeat gait at a particular speed, and thus, the comparison of parameters of particular gait cycle stages is more distorted. The method using the Zebris is more precise in this respect.

Another study of Sedaghati et al. (2016) reported improved functional postural stability and reduced fall risk after 10 weeks of exercise on a balance pad. A study of Nardo et al. (2014) reported improved UPDRS III scores after robotic-assisted training. This suggests favorably that gait training using a Zebris treadmill, which can operate in a training mode that is set up according to the outcome of initial treadmill examination in a particular patient, could have a positive effect on gait.

In a quantitative study, Vallabhajosula et al. (2015) reported that UPDRS III score, step length, and speed improved significantly during stimulation at 60 Hz and at > 100 Hz in contrast to no stimulation. Further increase in the stimulation frequency over 100 Hz did not result in further improvement. An increase in the stride length was also found in our patients under stimulation at 130 Hz.

A systematic study of Bakker et al. (2004) summarizing findings from nine studies showed a positive effect of DBS on postural instability and gait disturbances as assessed using UPDRS during the first year after surgery (Pötter-Neger and Volkmann, 2013). According to these studies, there was an increase in step length, which is in line with our results. In contrast to our results showing a small decrease in gait cadence (Figure 4), there were no changes in cadence in the systematic study (Bakker et al., 2004).

The evaluation of postural instability utilizes the questionnaire-based UPDRS. Other employed methods, such as those used in an article by Nutt et al. (2011), include quantitative assessment using the Tinetti Mobility Index, Berg Balance Scale, Balance Evaluation Systems Test, and Activities of Balance Confidence.

In our cohort of patients treated with bilateral STN-DBS, we observed improvements in cadence and step length after the stimulation initiation. The same result was reported in a study of Mazzone et al. (2014), in which the pedunculopontine nucleus was stimulated. It can be assumed that stimulation of both regions (i.e., the STN and the pedunculopontine nucleus) has a positive impact on gait and leads to its improvement.

According to the article by Nutt et al. (2011), changes in postural stability, and therefore in gait, in response to dopaminergic treatment and DBS treatment are ambiguous; in some patients, there is an improvement; in others, the effect is absent; in some other patients, the signs even worsen.

In this prospective study, we did not find a statistically significant correlation between the measured biomechanical gait parameters and patient age and disease duration. So it seems that positive effects of DBS on gait appear independent of age and disease duration.

What we see as a limitation of our study is gait examination under laboratory conditions when external influences are excluded (e.g., the need to go around furniture, walking down a busy street, poor visibility, etc.). An analysis by Warmerdam et al. (2020) points out different results of evaluated mobility parameters under laboratory conditions and in “everyday life.” When repeatedly analyzing unsupervised gait, we would see a limitation in comparison of gait at various speeds when parameters depending on speed, such as double-stance phase and cadence, are changing. On the other hand, it is important to analyze gait under conditions in which the patient moves in reality. It would be suitable to further analyze unsupervised gait, including gait speed, in future research and subsequently compare it with supervised gait under laboratory conditions with a moving treadmill set at a particular speed.

Further, it would be appropriate to include gait examination in the STN-DBS OFF state in future research too.

## Conclusion

Based on the results of the current study conducted within the past 4 years and 9 months, and which included 20 probands (all available participants), it can be concluded that DBS treatment in PD patients at the stage of late motor complications has a positive effect on gait as compared to dopaminergic treatment alone. We observed prominent clinical gait improvement in PD patients after DBS treatment initiation. The relevance of the results is supported by the methodological approach used, which enables an accurate assessment of biomechanical parameters of gait. The gait examination using a Zebris treadmill can be further employed in the subsequent use of the gait training system. Despite the relatively small number of probands in the conducted study, we consider the results to be significant because of prospective systematic data collection, the comparison with the baseline data before the implementation of DBS (rather than switching off the stimulation), and with respect to the precise analysis of the gait using the Zebris treadmill (comparing the biomechanical parameters of gait at exactly set speed, knowing that the parameters are changed according to speed of gait).

As we mentioned, prospective data collection is a lengthy process due to the small number of patients indicated for DBS therapy. The relatively low number of patients involved in the study is caused also by strict indication criteria and impossibility to include patients with reimplanted discharged stimulators and with implanted stimulation in the past. Despite modest sample size, the research results can be considered relevant.

## Data Availability Statement

The datasets generated for this study are available on request to the corresponding author.

## Ethics Statement

The studies involving human participants were reviewed and approved by the Ethics Committee of the Department of Neurology, Palacký University. The patients/participants provided their written informed consent to participate in this study.

## Author Contributions

DK performed surgery in all recruited patients and critically read the manuscript. DN substantially contributed to data acquisition, analysis, and interpretation and drafted the manuscript. AK substantially contributed to the design of the work. MN and PO substantially contributed by performing electrophysiological preoperative monitoring and postoperative care. PKa substantially contributed to the conception of the work and substantially revised the manuscript. PKo and BK worked on the study design in the gait laboratory and critically read the manuscript. MK, KM, MV, and SK participated in the study design and examined the patients. All authors have read, revised critically and approved the final submitted manuscript, and agreed to be accountable for the content of the work.

## Conflict of Interest

The authors declare that the research was conducted in the absence of any commercial or financial relationships that could be construed as a potential conflict of interest.

## Abbreviations

DBS: deep brain stimulation
fMRI: functional magnetic resonance imaging
*p*: *p* value
PD: Parkinson’s disease
STN: subthalamic nucleus
UPDRS: Unified Parkinson’s Disease Rating Scale.
